# Neuroplasticity in Motor Learning Under Variable and Constant Practice Conditions—Protocol of Randomized Controlled Trial

**DOI:** 10.3389/fnhum.2022.773730

**Published:** 2022-03-17

**Authors:** Stanisław H. Czyż, Jarosław Marusiak, Patrícia Klobušiaková, Zuzana Sajdlová, Irena Rektorová

**Affiliations:** ^1^Faculty of Physical Education and Sports, Wrocław University of Health and Sport Sciences, Wrocław, Poland; ^2^Faculty of Sport Studies, Masaryk University, Brno, Czechia; ^3^Physical Activity, Sport and Recreation (PhASRec), Faculty of Health Sciences, North-West University, Potchefstroom, South Africa; ^4^Department of Kinesiology, Faculty of Physiotherapy, Wrocław University of Health and Sport Sciences, Wrocław, Poland; ^5^Applied Neuroscience Research Group, Central European Institute of Technology, Masaryk University, Brno, Czechia; ^6^First Department of Neurology, Faculty of Medicine, Masaryk University, and St. Anne’s University Hospital, Brno, Czechia; ^7^Surgeon General Office of the Slovak Armed Forces, Ružomberok, Slovakia

**Keywords:** sensorimotor cortex activity, neuroplasticity, specificity of practice, variability of practice, practice conditions, motor learning

## Abstract

**Background:**

There is numerous literature on mechanisms underlying variability of practice advantages. Literature includes both behavioral and neuroimaging studies. Unfortunately, no studies are focusing on practice in constant conditions to the best of our knowledge. Hence it is essential to assess possible differences in mechanisms of neuroplasticity between constant vs. variable practice conditions. The primary objectives of the study described in this protocol will be: (1) to determine the brain’s structural and functional changes following constant and variable practice conditions in motor learning (structural and functional magnetic resonance imaging, MRI); (2) to determine the EEG activation and connectivity between cognitive, sensory, and motor cerebral cortex areas (central, temporal, parietal, occipital) in constant and variable practice conditions and as a function of practice time.

**Methods:**

The study will follow the interventional (experimental) design with two arms (parallel groups). Fifty participants will be randomly assigned to two groups practicing in constant (CG) and variable conditions (VG). CG will be practicing only one pattern of step isometric contractions during unimanual index finger abduction, i.e., 90 trials in all training sessions, whereas VG will practice three different patterns. Each will be practiced 30 times per session in variable conditions. Resting-state fMRI, EEG (cortical networking), and motor task proficiency will be examined before (pre-) and after practice (post- and retentions tests).

**Discussion:**

Findings will enhance our understanding of structural and functional neural changes following practice in constant and variable conditions. Therefore, the study can be considered pure (basic) research (clinical research in healthy individuals).

**Clinical Trial Registration:**

Study registered at clinicaltrials.gov (ID# NCT04921072) on 9 June 2021. Last version update: 21 December 2021.

The protocol has been prepared according to the complete SPIRIT checklist (http://www.spirit-statement.org/), although the item order has been modified in order to comply with the manuscript structure.

## Introduction

### Background and Rationale

The problem of efficiency in motor learning under variable and constant practice conditions has been addressed in many research for many years, e.g., it has a special place in Schmidt’s schema theory (Schmidt, 1975, 2003). The theory assumes that practicing many variations of skill develops memory representation (motor schema) better. As a result, a better-developed schema positively affects the transfer of a skill performed in a novel situation.

We also know that variable practice involving the practice of several variations of a skill benefits learning differently than practice in constant conditions, i.e., a practice that involves only one variation of a skill (Czyż, 2021; Kim et al., 2021b). The variable practice results in better retention and transfer (Schmidt and Lee, 2011). The performance of a skill practiced in variable conditions is more accurate and stable (Keetch et al., 2005; Breslin et al., 2012). In contrast, practicing only one variation of a skill better refines recall schema (Keetch et al., 2005; Breslin et al., 2012). It means that the motor program (which serves as an, “example,” while executing a movement) is developed better (Keetch et al., 2005). The trained variation of a skill (in constant practice) produces an advantage in performance as compared to the same variation of the skill that was practiced in variable conditions (assuming that variable and constant practice had similar capacity; Schmidt and Lee, 2011). This finding has an important implication for those who want to master their skills. If one wants to be good at performing only one variation of skill, one should practice in constant conditions. In contrast, if one wants to be good in more than one variation of skill and generalize the experience to novel situations, an individual should practice in variable conditions (Czyż and Moss, 2016). As one can see, this implication is practical, although the mechanisms underlying this distinction and differences are unknown.

It is unquestioned today that learning new motor skills dynamically changes the brain structure and function, i.e., the brain is neuroplastic (Mang et al., 2020). The neuroplasticity of the brain is specifically conspicuous in the progression of motor learning. As it was reported in previous research, cortico-striatal and cortico-cerebellar systems play an essential role in motor skill acquisition (Doyon et al., 2009; Lohse et al., 2014). However, both of these systems differ in terms of their role as the learning progresses. Cortico-striatal system (associative/premotor brain regions) is primarily engaged in the initial phase of learning, i.e., in cognitive functioning and sensory processing. The cortico-striatal system consists of the dorsolateral prefrontal cortex, rostral premotor areas, inferior parietal cortex, cerebellar cortex, and rostral basal ganglia (Lohse et al., 2014).

On the other hand, the primary motor cortex, supplementary motor area, dentate nucleus of the cerebellum, and putamen, are becoming more active in the later phase of motor learning. These brain regions constitute cortico-cerebellar system (sensorimotor network; Mang et al., 2020). It has been hitherto evinced that the brain structure changes due to motor learning. There was an increase in gray and white matter mass after a new motor skill (bimanual three-ball juggling) was practiced (Draganski et al., 2004, 2014; Boyke et al., 2008). Structural changes are noticeable in MRI, although none of the previous research focused exclusively on practice conditions. Moreover, none of the previous research focused on what role these systems play in learning under different conditions and how the different roles the systems may play affect the structural neuroplasticity, including gray and white matter. Therefore, we decided to use MRI to assess whether and what changes in brain structures follow skill practicing in constant and variable conditions.

Apart from potential structural differences, functional neuroplasticity will also be assessed. A lesser degree of cognitive involvement during movement execution may be associated with lower activation in the sensorimotor cortex (Cheng et al., 2015). On the other hand, increased cognitive involvement may be expected in variable conditions due to, e.g., due to stimulus identification or decision making. Therefore, an assumption that decreased cognitive involvement and, as a result, decreased prefrontal cortex activation in constant practice conditions sounds reasonable. Moreover, we may hypothesize that practicing and learning in constant conditions will be characterized by lower sensorimotor cortex activation since there will be decreased control during the motor performance, which leads to more adaptive motor performance. Vernon and colleagues (Vernon et al., 2003) showed that in order to reduce the somatosensory interference in information processing, their participants enhanced SMR power as indexed by increased SMR/theta and SMR/beta ratios. As a result, participants were able to maintain perception and, to a lesser extent, focused attention. Assuming that parametrization has to take place under variable practice conditions every time one changes performance from one variation of skill to another, demand on cognitive resources (perception and attention) would be bigger than in constant conditions, in which parametrization is facilitated. Consequently, the variable practice group should increase SMR as compared to the constant practice conditions group.

There have been no previous studies on cortical networking in constant and variable practice and motor learning; we decided to assess such communication measuring EEG coherence (Deeny et al., 2009). A significant linear relationship exists between time series simultaneously recorded from two locations (high coherence), indicating communication between these areas of the cerebral cortex. In contrast, low coherence indicates the opposite (Deeny et al., 2009). We may expect decreased coherence between premotor (Fz) and motor (C3, C4) areas of the cortex and between the premotor and occipital regions as the practice of a visuo-motor task progresses (Busk and Galbraith, 1975). However, if the constant and variable practice condition groups receive the same amount of practice, we may expect that pairing electrodes in specific regions will be undifferentiated (Deeny et al., 2003). Lelis-Torres and colleagues (Lelis-Torres et al., 2017) noted that when investigating the cognitive effort involved in random compared to constant practice schedules, the random practice induced significantly greater cognitive effort than constant practice. Throughout the practice, both task engagement and mental workload decreased more in the constant practice condition than in the random practice condition. However, the random schedule is only one variant of variable practice. Another extreme is the blocked practice schedule. Variable practice in blocked order assumes that a learner is practicing many skill variations but in blocks (or time). Although, authors agree that this is also variable practice (Van Rossum, 1987; Czyż, 2021), some doubt whether this variability differs from constant practice (Kim et al., 2021b). This ambiguity will be tested in our study.

### Objectives

The primary objectives of our study are:

1. To determine functional brain changes following (neural underpinnings of) constant and variable practice conditions in motor learning (resting-state fMRI).

2. To determine the EEG activation and connectivity between cognitive, sensory, and motor cerebral cortex areas (central, temporal, parietal, occipital) in constant and variable practice conditions and as a function of practice time.

### Trial Design

**Figure 1:**
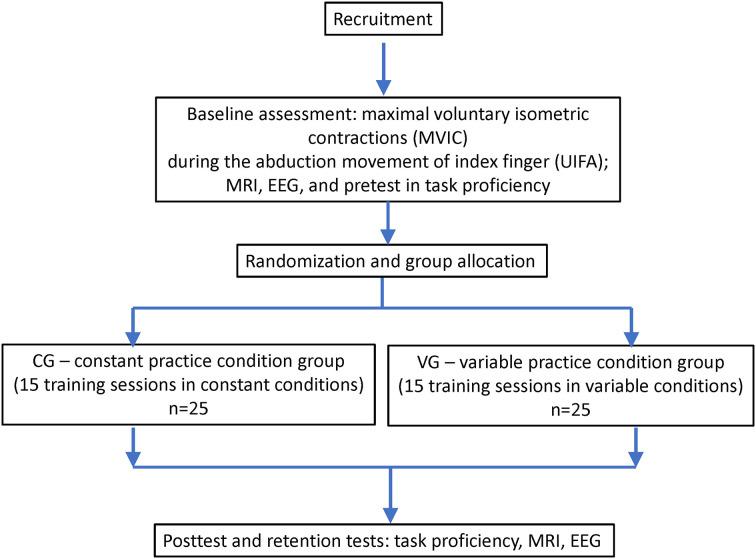
Flowchart of the study.

The study will follow the interventional (experimental) design with two arms (parallel groups). The study protocol was approved by Masaryk University Research Ethics Committee (EKV-2021-057) and was registered at clinicaltrials.gov (ID# NCT04921072). The outline of the study design is shown in Figure 1.

## Methods

### Participants, Interventions, and Outcomes

#### Study Setting

The study will be conducted at Multimodal and Functional Imaging Laboratory (MAFIL), Masaryk University, Brno, Czechia, and at CEITEC and Faculty of Sport Studies, Masaryk University, Brno, Czechia.

### Eligibility Criteria

#### Inclusion Criteria

Young, healthy male and female adults (20 years old–35 years old) will be recruited. Inclusion criteria: no history of epilepsy, any known neurological disorder, no psychiatric history, were medication-free during the previous 14 days prior to participation, had not used alcohol within the previous 24 h and were not pregnant (Kim et al., 2021a). Participants will be excluded if they were a musician or a professional typist, or had any contraindications to MRI, significant medical conditions that prevent them from performing the task (Lin et al., 2018), or scored less than three on the Mini-Cog^TM^ test (Borson et al., 2003).

Participants’ handedness will be determined using the German version of the Edinburgh Handedness Inventory (EHI) questionnaire (Oldfield, 1971; Loffing et al., 2014).

#### Exclusion Criteria

Participants who do not meet the inclusion criteria will be excluded. Additional exclusion criteria for the MRI experiments will be claustrophobia, pacemaker, and ferromagnetic metal material in the body. Participants declaring any contraindications for physical activity or being unhealthy will be excluded from the study.

#### Recruitment

A total of 50 participants will be recruited. We will address potential participants (whatever contact is preferable) from the already existing databases at CEITEC with people willing to be contacted and willing to participate in the neuroimaging projects. We will also utilize participants invited by doctoral students involved in the project. These will identify further potential participants.

### Interventions

#### Explanation for the Choice of Comparators

The constant practice condition group will be used as a comparator. Although, one could choose the variable practice conditions (in blocked form) group, as well. They, i.e., constant and variable practice conditions, are but two extremes of one continuum (Czyż, 2021). Therefore, our choice is arbitrary (comparator could be either extreme).

#### Intervention Description

##### Motor Task

**Figure 2:**
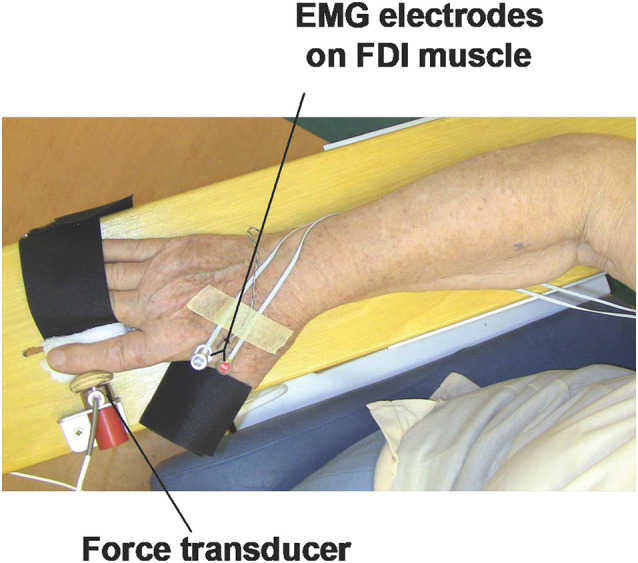
Set-up for the unimanual index finger (UIFA) abduction motor task.

The participants will be performing unimanual (with dominant right hand) index finger abduction (UIFA) using custom-made equipment (Figure 2) consisting of:

***(a)** wooden table/board* to support forearm during testing;

***(b)** force transducer* to measure isometric force during UIFA motor task; the force transducer (WMC, Interface, Arizona, USA) used in our device is highly sensitive to levels of generated isometric force during the UIFA motor task (force measurement range 0–120 N); the output force signal of the transducer will be filtered and amplified with *SGA conditioner* (Interface, Arizona, USA), and then directed to a *multichannel CED card* (Cambridge Electronic Design, Ltd., Cambridge, UK) and recorded using *Spike* 2 s*oftware, version 7.10* (Cambridge Electronic Design, Ltd., Cambridge, UK). The sampling frequency of simultaneously recorded force signals will be 2,048 Hz;

***(c)** wooden button connected to the force transducer* to transfer a force generated by index finger to force transducer; the wooden button is shaped to arched shape of the region of middle phalanx of index finger; and *small mounting metal and plastic elements* (laminas, nuts, and screws) used to connect elements to each other and to attach elements to the wooden table/board;

***(d)** two supporting Velcro straps* to stabilize fingers that are not engaged in UIFA motor task; the first strap will stabilize the thumb finger, and the second strap will stabilize the third to fifth fingers (in the tested hand); this way, we will isolate the abduction movement of the index finger (UIFA);

First, the participants will be asked to perform three 5 s ongoing trials of maximal voluntary isometric contractions (MVIC) during the UIFA (MVIC-UIFA), separated by 3 min rest intervals. The MVIC-UIFA force value will be then obtained as an average value calculated from the three MVIC-UIFA trials.

**Figure 3:**
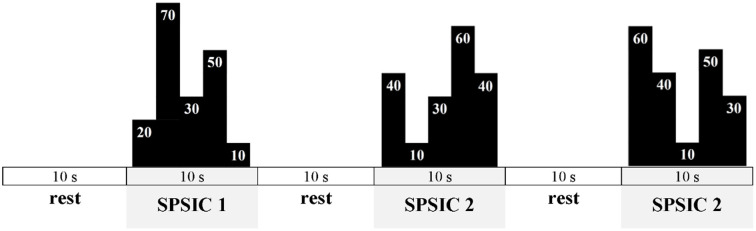
Exemplary scheme of the motor task displayed during tests and training. The final values of UIFA task during each SPSIC are not shown as we would like to ensure the novelty of the task to the participants.

Based on the MVIC-UIFA force value, an automatic algorithm of UIFA force feedback system (implemented in the CED Spike2 environment) will be able to set automatically the percentage submaximal level of force to be achieved by tested participants while performing various specific patterns of step isometric contractions (SPSIC) during UIFA. Each SPSIC will be a sequence of five consecutive different percentage UIFA force steps (the range of percentage steps: 10%, 20%, 30%, 40%, 50%, 60%, 70%, 80%, and 90% of MVIC-UIFA force) to be achieved by the participants (as presented with black schemes on Figure 3).

Each of the five steps of a single SPSIC will be reached as fast as possible and sustained for 2 s, which gives a 10 s performance time for each SPSIC. Participants will perform SPSIC with 10 s rest intervals between each SPSIC (as in Figure 3).

CG and VG will differ in terms of practiced schemes. CG will be practicing only one SPSIC scheme: SPSIC 1. It means that 90 trials in all training sessions will consist only of SPSIC 1. On the other hand, VG will practice three SPSIC’s (1–3). Each SPSIC will be practiced 30 times per session in variable conditions, which means that each session will consist of 90 SPSIC like in CG.

Thus, taking together the performance time of 90 SPSIC’s (90 SPSIC’s × 10 s = 900 s) and 90 10 s lasting rest intervals (900 s in total), the whole SPSIC motor task performance will last about 1,800 s ≈30 min. After adding the MVIC-UIFA’s performance time (3.5 min), the whole UIFA motor task performance time during the testing session will be about 35 min. The total number of trials and time of training sessions will be the same in both groups, which is consistent with the previous variability of practice study design (e.g., McCracken and Stelmach, 1977). There will be one training session each working day for 3 weeks, which means each participant will accumulate 15 training sessions in total, 1,350 trials. Given the structural changes in MRI (specifically DW-MRI) may be conspicuous after a 3 week discrimination training based on Braille reading (Debowska et al., 2016), we assumed that 3 weeks of training should be enough to observe any structural changes in both groups.

On the other hand, structural changes in gray matter were observed after several hours of training in jugglers in a study by (Draganski et al., 2004). They performed scans in participants who were able to bimanually juggle with three balls for at least 60 s and noticed structural changes in the fusiform gyrus, the medial frontal gyri, and the inferior parietal lobule. Although they did not provide when participants achieved the targeted juggling time (i.e., 60 s), it may be assumed it did not take long. It usually takes hours or days to juggle with three balls (Beek and Lewbel, 1995). In experiments by Knapp and Dixon (1952), participants with no previous juggling experience were able to make 100 consecutive catches after 65–77 trials on average. On the other hand, 3 week training is considered as a long-term learning (Floyer-Lea and Matthews, 2005). Therefore, we assumed that 3 weeks of training should be sufficient to induce structural changes in the brain after our training program.

### Criteria for Discontinuing or Modifying Allocated Interventions

Participants who will not participate in all training sessions will be excluded from the study. The obtained will be deleted.

### Strategies to Improve Adherence to Interventions

Participants will be contacted on a daily basis in order to confirm their availability and willingness to participate in the study.

### Relevant Concomitant Care Permitted or Prohibited During the Trial

There are no relevant concomitant care or interventions that are permitted or prohibited during the intervention.

### Outcomes

Primary Outcome Measure:

1. Brain structural and functional changes following constant and variable practice conditions in motor learning (structural, diffusion, and resting-state fMRI):

Structural, diffusion, and resting-state functional scans will be acquired both prior to and after the 3 weeks of practicing the unimanual index finger abduction motor task. High-resolution T1-weighted (MPRAGE) and FLAIR images will be exploited to assess gray matter changes. Diffusion-weighted (DWI) data will be used for probabilistic tractography to obtain specific tracts that will be analyzed in terms of alterations in fractional anisotropy, mean, radial, and parallel diffusivity. In addition, whole-brain white matter microstructural changes will be assessed using Tract-Based Spatial Statistics (TBSS).

Regarding resting-state fMRI data, BOLD multi-echo echo-planar imaging fMRI sequence will also be performed. In addition to resting-state functional connectivity (rs-FC) analysis among regions of interest, an Independent Component Analysis will also be used to evaluate rs-FC changes of large-scale brain networks of interest.

2. EEG brain waves characteristics and motor task proficiency outcomes (estimated from simultaneous EEG and force recordings during motor task performance, i.e., UIFA) differences in constant and variable practice conditions in motor learning and as a function of practice time:

To record bioelectrical signals from the brain cortex (EEG) during the performance of unimanual index finger abduction (UIFA) motor tasks, we will use the scalp 256-channel EEG data acquisition system (Electrical Geodesics, Inc., Eugen, OR, USA) with sponge electrodes (the “wet” EEG system by Electrical Geodesics, Inc. Eugen, OR, USA). The force induced during the UIFA will be recorded and visualized for participants (as feedback) using the same custom-made system as described in the Section “Intervention Description”. Also, during the UIFA performance, the same force signal will be sent synchronously to the EEG acquisition system (Electrical Geodesics, Inc., Eugen, OR, USA), and will be recorded simultaneously. The force signals will be used as triggers for subsequent EEG analysis.

Two types of UIFA motor paradigms will be used during simultaneous EEG and force recordings in pre- post-, and retention tests, i.e., (1) the UIFA SPSIC paradigm (SPSIC—step isometric contractions) and (2) UIFA MRCP paradigm (MRCP—motor-related cortical potential).

The participants from VG will perform three types of SPSICs (1–3), each repeated 15 times (together 45 trials), but the participants from CG will perform 45 times of one type of SPSIC. This SPSICs will be analyzed in the frequency domain using force signal as a trigger for extracting SPSIC sequences related to time frames of EEG time series data. We will estimate EEG signal’s power spectrum density (PSD) for sensorimotor rhythm (SMR, frequency band 12–15 Hz) as well as for other frequency bands: delta (0.5–4 Hz), theta (4–8 Hz), alpha (8–14 Hz), beta (14–35 Hz) and gamma (35–70 Hz). PSD-EEG analysis will reveal the level of the electrical activity of cortical regions engaged during UIFA motor tasks performance under the constant and variable practice conditions. We will also estimate coherence between EEG signals (EEG-EEG coherence) from electrodes in a chosen region of interest based on the PSD-EEG analysis. Analysis of EEG-EEG coherence calculates the linear relationship between the power spectra of two EEG signals taken from two EEG electrodes placed on the scalp. A significant linear relationship existing between time series simultaneously recorded from two locations (high coherence) indicates a high level of functional communication (connectivity) between these areas of the cerebral cortex, whereas low coherence indicates the opposite.

Both groups will perform 40 MRCP trials, i.e., 10 s radial abduction to 20% MVC of UIFA task and 10 s rest. The MRCP will provide a reliable time-domain estimation of the amplitude, latency, source, and connectivity analysis. The EEG signals recorded during the MRCP paradigm will be analyzed using: (1) averaging technique to assess an amplitude of MRCP showing the level electrical activity of cortical regions engaged during motor task performance, (2) EEG source analysis, showing current dipole of activation as a model of the electrical current, that characterizes of location, orientation and strength or amplitude [expressed in dipole moment (nAm)], which describes the directional activity of brain region engaged in a motor task, and (3) time-frequency and connectivity analysis, to explore the strength of functional coupling between cortical regions responsible in motor preparation and execution. We will analyze the EEG data in co-registration with individual T1 structural MRI scans in the Talairach space, a 3-dimensional human brain coordinate system used to map the location of brain structures independent from individual differences in the size and overall shape of the brain.

UIFA motor task proficiency outcomes for the UIFA SPSIC and UIFA MRCP will be obtained based on the force signal processing and analyses in the time domain. We will estimate a motor performance accuracy based on the error percentage of achieved force level in reference to the given target force level while performing the various sequences of SPSIC-UIFA motor tasks. Also, we will assess the speed of force generation by estimating the time and rate of force development during the execution of the various sequences of SPSIC-UIFA motor tasks.

### Participant Timeline

The schedule of enrolment, interventions, and assessments is presented in Table 1 (according to SPIRIT Statement).

**Table 1:** Schedule of enrolment, interventions, and assessments.

	**Study period**					
	**Enrolment**	**Allocation**	**Post-allocation**			**Close-out**
**Timepoint**	***Week 0***	Week 0	***Week 1***	***Week 2***	***Week 3***	***5-days retention test (Week 4)***
**Enrolment**				
Eligibility screen	X				
Informed consent	X				
Allocation		X			
**Interventions**				
*Constant practice*			X	X	X
*Variable practice*			X	X	X
**Assessments**				
*Demographic information*	X	X			
*EEG*		X			X	X
*MRI*		X			X	X
*Motor task proficiency*		X	X	X	X	X

### Sample Size

In order to calculate the sample size, we used mean and standard deviation (SD) values (Dean et al., 2013) presented by Cheng and colleagues in Table 1, specifically, values for Time Window 1 (T1) for novice and expert participants (Cheng et al., 2015). We set confidence interval = 95%; power = 0.8; ratio of sample size = 1 (constant practice group vs. variable practice group). As a result, a minimal sample size estimated was eight participants in total, four participants per group. Given, we were going to recruit novices for both groups, the possible drop-out, statistical requirements (e.g., at least five observations perc variable in regression models), and considering potential recording problems, we decided to measure 25 participants per group.

### Recruitment

A total of 50 participants will be recruited. We will address potential participants (whatever contact is preferable) from the already existing databases at CEITEC with people willing to be contacted and willing to participate in the neuroimaging projects. We will also utilize participants invited by doctoral students involved in the project. These will identify further potential participants (snowball). Flyers and posters will be disseminated on the university campus and surrounding public places.

### Assignment of Interventions: Allocation

#### Sequence Generation, Concealment Mechanism, and Implementation

All participants will be randomly assigned to the constant and variable practice condition groups (CG and VG, respectively) with a 1:1 allocation ratio. An online randomization generator^1^ with three blocks of four, three blocks of six, and two blocks of 10 participants will be used.

### Assignment of Interventions: Blinding

#### Who Will Be Blinded

Given the nature of the intervention and the objectives of the study (basic science), neither participants nor the researchers will be blinded.

### Data Collection and Management

#### Plans for Assessment and Collection of Outcomes

Pretest, posttest, and retention tests will consist of MRI, EEG, and motor task proficiency assessments.

Changes in the gray matter will be estimated using voxel-based morphometry (Ashburner and Friston, 2000), which constitutes the whole-brain gray matter probabilities comparison. In addition, volumetric analyses will be calculated on both cortical and subcortical structures, and cortical thickness can be evaluated (Fischl et al., 2002). Variations in white matter microstructure can be assessed using Tract-Based Spatial Statistics (TBSS; Smith et al., 2006)—a method that applies voxel-wise statistics on diffusion metrics using projection onto an alignment-invariant tract representation. Representations of specific tracts can be obtained with probabilistic tractography (Behrens et al., 2007) that takes into account crossing fibers in individual voxels. The BOLD multi-echo echo-planar imaging (ME EPI) fMRI sequence will be applied regarding resting-state fMRI data. Representative time-series within chosen regions of interest (ROIs), i.e., dorsolateral prefrontal cortex, posterior parietal cortex, posterior parietal cortex, primary motor cortex, anterior cingulate cortex, cerebellar cortex, cerebellar dentate, putamen, thalamus primary, and motor cortex, will be extracted and used for seed-based analysis computation. Moreover, Independent Component Analysis (ICA) will be calculated in order to evaluate changes in large-scale brain networks’ connectivity, with the emphasis on fronto-parietal control network, dorsal attentional network, basal ganglia network, and sensorimotor network.

In order to test the sensorimotor cortex activity in constant and variable practice conditions, we decided to record the sensorimotor rhythm (SMR), which oscillates between 12 and 15 Hz in the motor cortex. SMR is widely used to indicate the activity of the sensorimotor cortex (Egner and Gruzelier, 2001, 2003) since there is a positive relationship between SMR power and inhibition of sensorimotor cortex activity.

Motor task proficiency will be measured as efficiency in repeating patterns of step isometric contractions (SPSIC) during UIFA using custom-made equipment.

### Plans to Promote Participant Retention and Complete Follow-Up

All assessments and training sessions will be scheduled by research assistants who will contact them prior to their visit.

### Data Management

Original EEG, demographic and personal data will be stored in a secure, MU password-protected repository. The original non-anonymized file will be password protected (two-phase authentication).

MRI safety forms are archived at a secured place at the MAFIL core facility. Electronic data from these forms is stored in a secured database (with two-phase authentication) by ICS MU. Original MRI data are stored in secure MEDIMED infrastructure (suitable for dealing with medical/healthcare data) provided by ICS MU.

### Statistical Methods

#### Statistical Methods for Primary and Secondary Outcomes

In order to compare groups practicing in constant and variable practice conditions in terms of brain’s structural and functional changes, repeated measures ANOVA with time as a within-participants factor, and practice condition as a between participants factor, with *post-hoc* tests will be used. The relationship between MRI brain changes and motor task proficiency will be evaluated using partial correlations.

Frequency analysis will be performed separately for each EEG channel and trial using fast Fourier transform (FFT). Significant differences against baseline will then be compared between participant groups. Connectivity analysis will be performed using the calculation of coherence (Nunez et al., 1997, 1999) between channels of interest. Results will be statistically compared between participant groups. Because of the multiple usages of statistical tests, multiple comparison corrections will be used.

Motor task proficiency will be assessed using parametrical or non-parametrical tests, depending on the results of the assumption tests (homogeneity, normality). The possible tests include but are not limited to correlations, t-test, and analysis of variance (2 × 3 model; two practice conditions × three time points) with the following *post-hoc* analysis for all significant main effects.

We may use Bayesian equivalents of the tests mentioned above.

### Interim Analyses

No interim analyses are planned.

### Methods for Additional Analyses (e.g., Subgroup Analyses)

No additional analyses are planned.

### Methods in Analysis to Handle Protocol Non-adherence and any Statistical Methods to Handle Missing Data

Participants with missing data due to the absence during tests or training sessions will be excluded from the study. The data will not be analyzed.

Participants with missing data will be excluded from further analysis.

### Plans to Give Access to the Full Protocol, Participant Level-Data and Statistical Code

All anonymized participant’s individual data will be shared using one of the many open repositories; however, how the data will be organized and how it will be presented has to be decided (given the complexity and amount of imaging data).

### Oversight and Monitoring

#### Composition of the Coordinating Center and Trial Steering Committee

Frequent weekly meetings will be organized. SC is the primary investigator (PI). The protocol manuscript was drafted by SC. IR—reviewed, edited, and supervised the protocol. She will be supervising MRI assessments and providing help in interpreting results. JM is responsible for the motor task description and administration, EEG measurements, and data analysis. PK is responsible for MRI description, administration, and data analysis. ML is responsible for EEG data acquirement quality. ZS is responsible for participants recruitment, randomization, and practice scheduling (participants management). Research assistants, including but not limited to doctoral students, post-docs, are responsible for participants management, providing and supervising training sessions, and scheduling and participants management.

#### Composition of the Data Monitoring Committee, Its Role, and Reporting Structure

There will be no monitoring committee. PI will be managing the team and the research.

### Adverse Event Reporting and Harms

Participants will indicate whether they wish to be informed about possible incidental findings in the informed consent form. It will be brought to participants’ notice that the research scans (MRI) or EEG analysis obtained in the current study are not part of any medical screening procedure, will not be evaluated by a trained physician, and are not intended to provide any information that may help in the diagnosis of any medical condition.

### Frequency and Plans for Auditing Trial Conduct

There will be no independent auditing.

### Plans for Communicating Important Protocol Amendments to Relevant Parties (e.g., Trial Participants, Ethical Committees)

Any amendments, including administrative amendments, will be approved by Masaryk University Research Ethics Committee prior to implementation. Amendments in the RCT registry will be applied accordingly.

### Who Will Take Informed Consent?

A team member, e.g., doctoral student, post-doc, or active researcher, will meet with prospective participants and explain the study’s objectives and all procedures and collect informed consent. If there was a dependent relationship between the team member and the potential participant (e.g., student—academic, patient—medical doctor), an independent person from Masaryk University would be asked to provide a short meeting to the potential participant(s). This independent person will be an active researcher with at least a doctoral degree. All of the issues of the dependent relationship will be discussed. Participants will be informed that they may resign at any time without any consequences.

### Additional Consent Provisions for Collection and Use of Participant Data and Biological Specimens

Not applicable—no biological specimens will be collected.

### Confidentiality

All data will be coded, and data processing will be performed on coded entries. The only file allowing to identify a participant personally will be kept in the PI’s computer. The identifying file will be kept for 5 years and will be anonymized afterward. The data from the MRI safety form and original MRI recordings containing personal data will be secured and processed by the authorized employee of the MAFIL facility. Pseudonymized (coded) data will be used for subsequent data processing.

### Availability of Data and Materials

The investigators will have access to the raw and final dataset. We disclose contractual agreements that limit such access for investigators.

### Provisions for Post-trial Care

Since our intervention does not harm anyone and no serious side effects are anticipated, there are no relevant provisions for ancillary or post-trial care.

### Dissemination Policy

Study results will be disseminated to academic and general communities. Dissemination means include but are not limited to publications, conference presentations, social media information.

### Plans for Collection, Laboratory Evaluation and Storage of Biological Specimens for Genetic or Molecular Analysis in This Trial/Future Use

Not applicable.

### Trial Status

Recruitment will start in January 2022. The current protocol version is 02, dated 21st December 2021 (the last update at clinicaltrials.gov).

## Discussion

There is pretty abundant literature on mechanisms underlying the variability of practice advantages. Previous studies specifically focused on differences between random and blocked practice effects (Kim et al., 2021b). However, both of them, i.e., random (also called interleaved practice) and blocked (also called repetitive practice) scheduled practice, are just variable practice. Literature about variable practice includes both behavioral and neuroimaging studies.

Unfortunately, no studies compare practice in constant and blocked (variable) conditions to the best of our knowledge. Although studies are comparing constant and variable practice in its random form, there is no proof yet that constant practice differs from blocked variable practice (Kim et al., 2021b). Hence it is vital to assess possible differences in neuroplasticity between constant vs. variable practice conditions in its blocked order.

Findings will enhance our understanding of structural and functional neural changes following practice in constant and variable conditions. Therefore, the study can be considered pure (basic) research. However, it may also have a practical implication. Assuming there will be a difference between constant and blocked practice, one could recognize different levels of practice variability based on, e.g., cognitive load related to different conditions. Consequently, these levels of variability (Van Rossum, 1987; Czyż, 2021; Kim et al., 2021b) could be manipulated in order to decrease/increase cognitive load during practice, potentially leading to different memory consolidation. Learners will be able to apply variability depending on the learning progress and objectives, e.g., decreasing cognitive load to enhance motor control mechanisms or increasing cognitive load to develop decision-making processes.

## Ethics Statement

The study protocol was approved by Masaryk University Research Ethics Committee (EKV-2021-057) and was registered at clinicaltrails.gov (ID# NCT04921072). All participants provided written informed consent to participate in the study. The study was conducted according to the Declaration of Helsinki and the Ethical Principles and Guidelines for the Protection of Human Subjects of Research (commonly called the Belmont Report), promulgated in 1979.

## Author Contributions

SC is the Principal Investigator, developed the first draft of the protocol and manuscript, applied for findings. JM is responsible for the training motor task description, administration, and supervising the EEG and force recordings and data analysis. PK is responsible for MRI data quality control and data analysis. ZS is responsible for participants recruitment and randomization, and practice scheduling (participants management). IR reviewed, edited and supervised the protocol. She will be responsible for MRI data interpretations. All authors contributed to the article and approved the submitted version.

## Conflict of Interest

The authors declare that the research was conducted in the absence of any commercial or financial relationships that could be construed as a potential conflict of interest.

## Publisher’s Note

All claims expressed in this article are solely those of the authors and do not necessarily represent those of their affiliated organizations, or those of the publisher, the editors and the reviewers. Any product that may be evaluated in this article, or claim that may be made by its manufacturer, is not guaranteed or endorsed by the publisher.

## Footnotes

^1^ http://www.randomization.com
